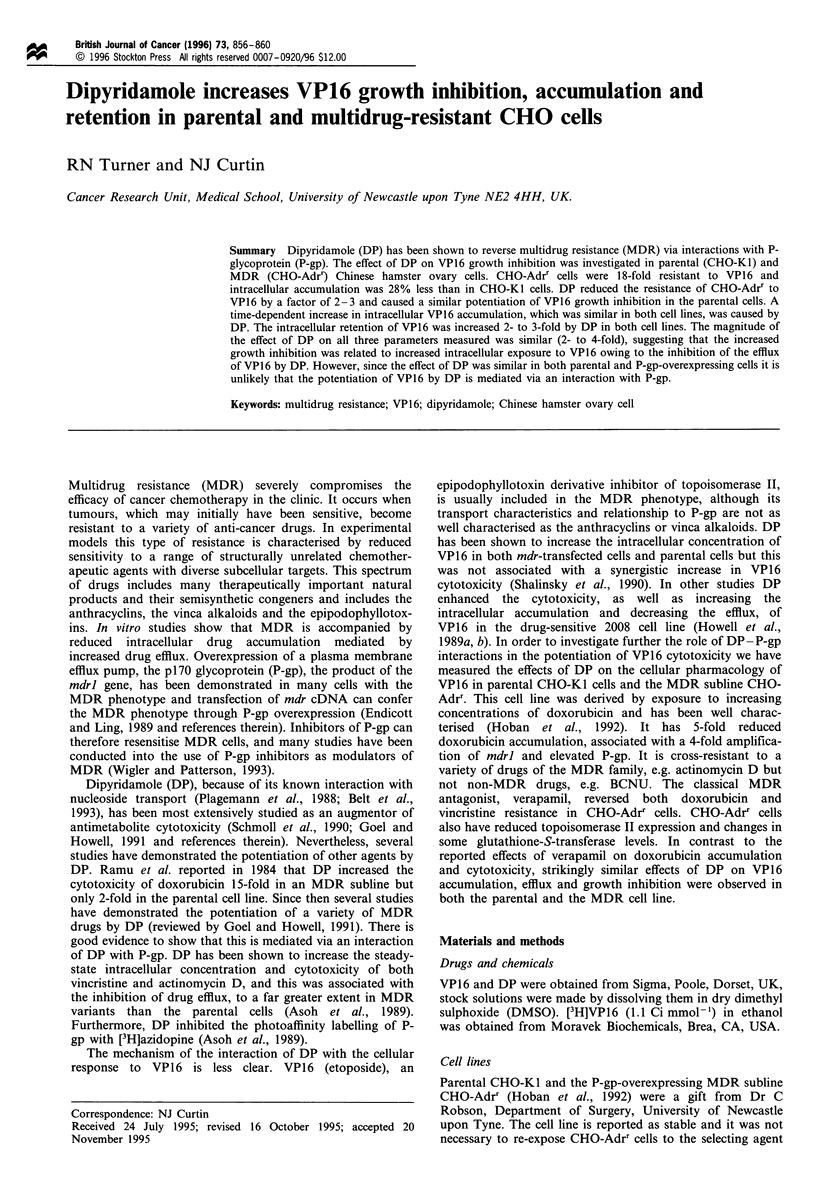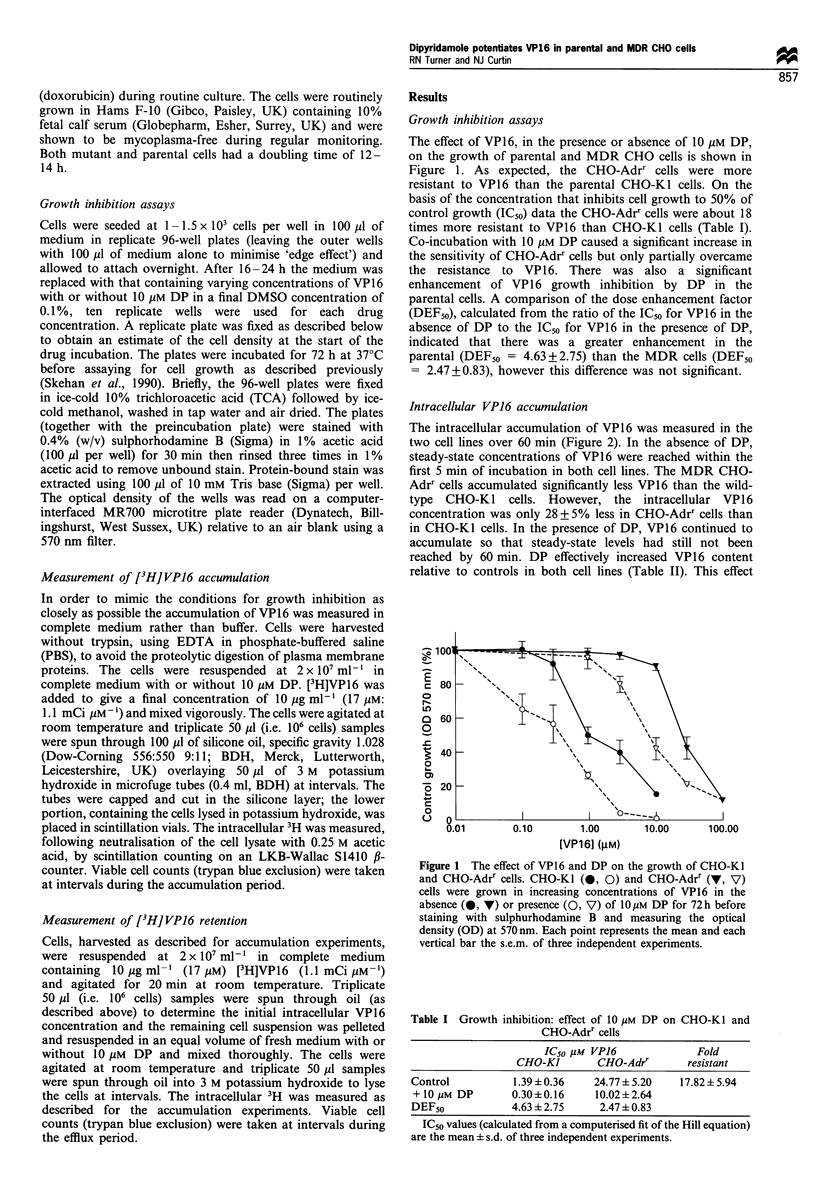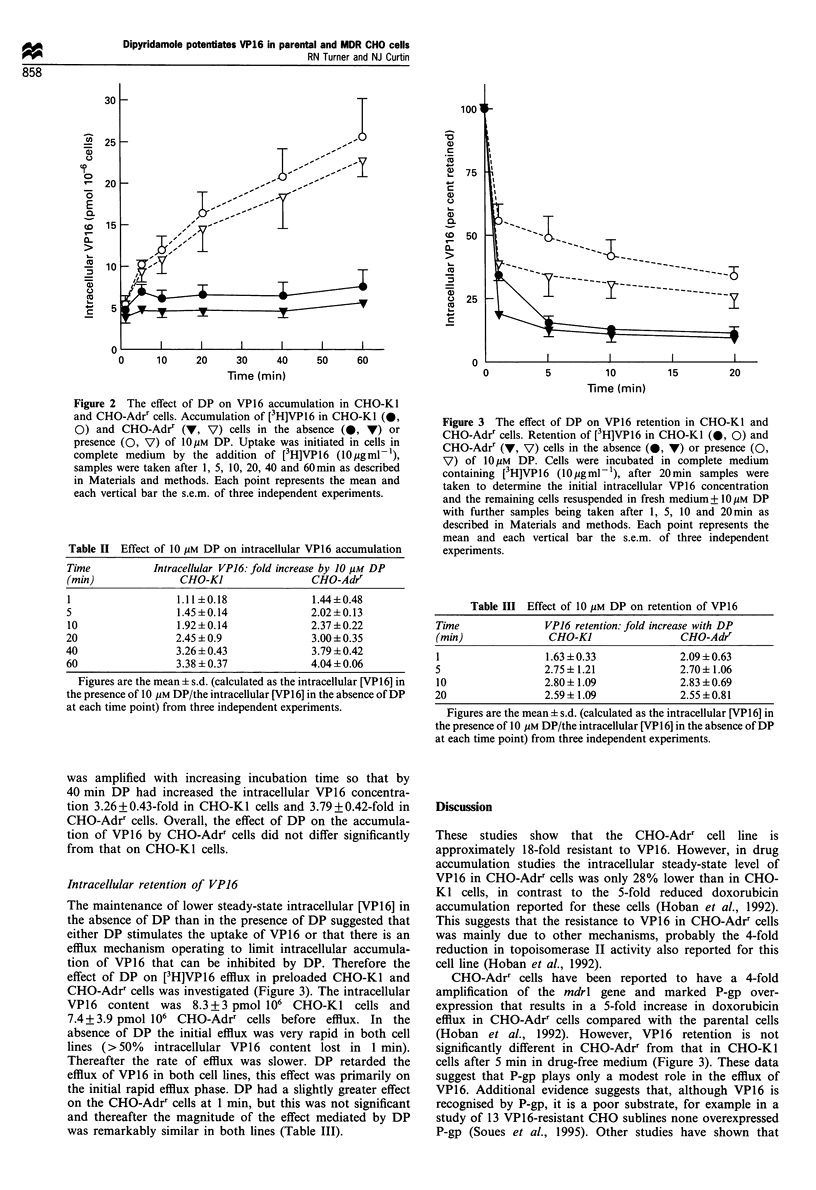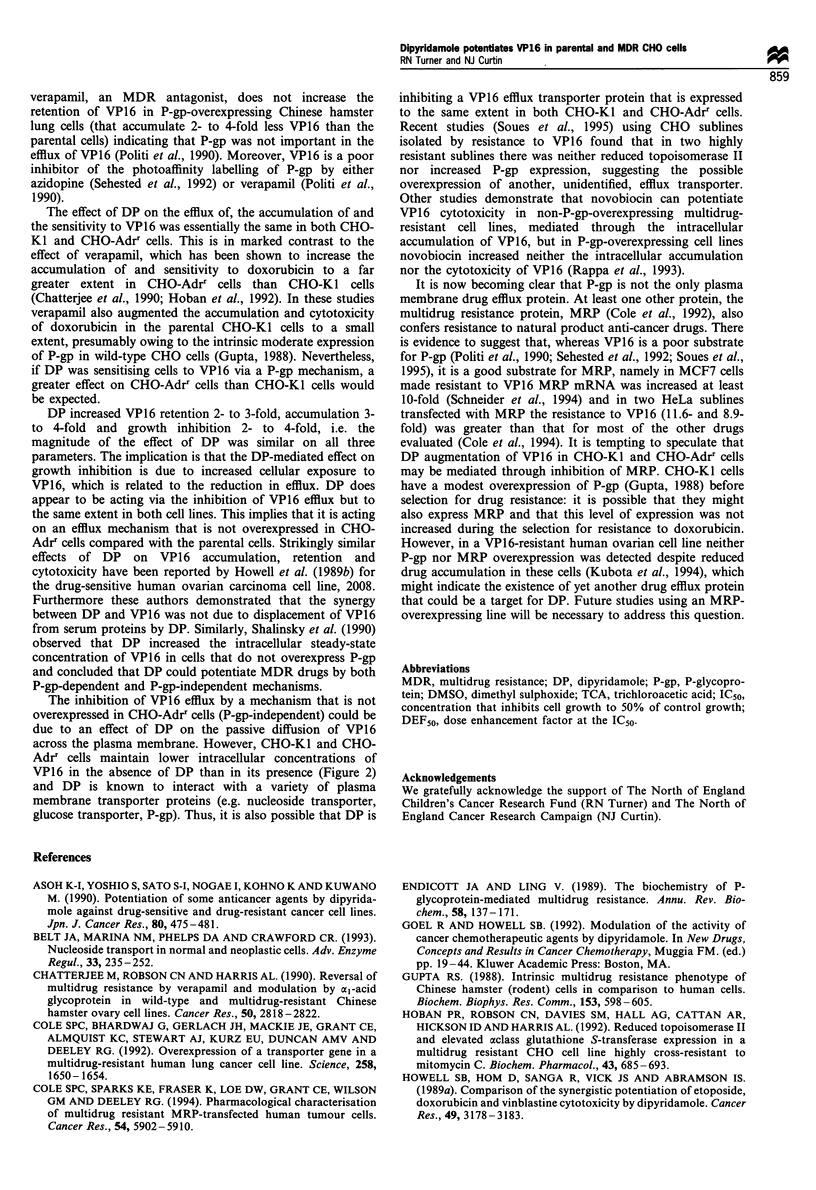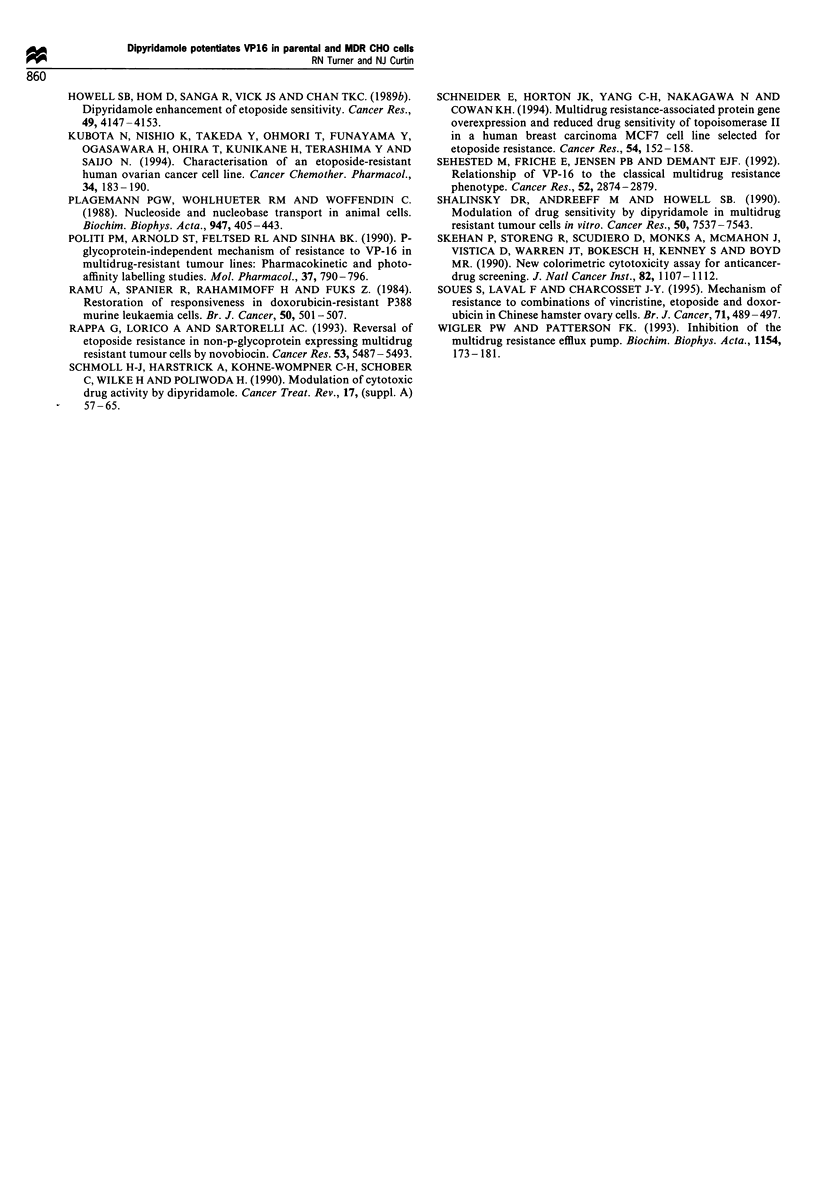# Dipyridamole increases VP16 growth inhibition, accumulation and retention in parental and multidrug-resistant CHO cells.

**DOI:** 10.1038/bjc.1996.152

**Published:** 1996-04

**Authors:** R. N. Turner, N. J. Curtin

**Affiliations:** Cancer Research Unit, Medical School, University of Newcastle upon Tyne, UK.

## Abstract

Dipyridamole (DP) has been shown to reverse multidrug resistance (MDR) via interactions with P-glycoprotein (P-gp). The effect of DP on VP16 growth inhibition was investigated in parental (CHO-K1) and MDR (CHO-Adr(r)) Chinese hamster ovary cells. CHO-Adr(r) cells were 18-fold resistant to VP16 and intracellular accumulation was 28% less than in CHO-K1 cells. DP reduced the resistance of CHO-Adr(r) to VP16 by a factor of 2-3 and caused a similar potentiation of VP16 growth inhibition in the parental cells. A time-dependent increase in intracellular VP16 accumulation, which was similar in both cell lines, was caused by DP. The intracellular retention of VP16 was increased 2- to 3-fold by DP in both cell lines. The magnitude of the effect of DP on all three parameters measured was similar (2- to 4-fold), suggesting that the increased growth inhibition was related to increased intracellular exposure to VP16 owing to the inhibition of the efflux of VP16 by DP. However, since the effect of DP was similar in both parental and P-gp-overexpressing cells it is unlikely that the potentiation of VP16 by DP is mediated via an interaction with P-gp.


British Journal of Cancer (1996) 73, 856-860
© 1996 Stockton Press All rights reserved 0007-0920/96 $12.00

### Dipyridamole increases VP16 growth inhibition, accumulation and retention in parental and multidrug-resistant CHO cells

RN Turner and NJ Curtin

*Cancer Research Unit, Medical School, University of Newcastle upon Tyne NE2 4HH, UK.*

**Summary** Dipyridamole (DP) has been shown to reverse multidrug resistance (MDR) via interactions with P-glycoprotein (P-gp). The effect of DP on VP16 growth inhibition was investigated in parental (CHO-K1) and MDR (CHO-Adr$^r$) Chinese hamster ovary cells. CHO-Adr$^r$ cells were 18-fold resistant to VP16 and intracellular accumulation was 28% less than in CHO-K1 cells. DP reduced the resistance of CHO-Adr$^r$ to VP16 by a factor of 2-3 and caused a similar potentiation of VP16 growth inhibition in the parental cells. A time-dependent increase in intracellular VP16 accumulation, which was similar in both cell lines, was caused by DP. The intracellular retention of VP16 was increased 2- to 3-fold by DP in both cell lines. The magnitude of the effect of DP on all three parameters measured was similar (2- to 4-fold), suggesting that the increased growth inhibition was related to increased intracellular exposure to VP16 owing to the inhibition of the efflux of VP16 by DP. However, since the effect of DP was similar in both parental and P-gp-overexpressing cells it is unlikely that the potentiation of VP16 by DP is mediated via an interaction with P-gp.

**Keywords:** multidrug resistance; VP16; dipyridamole; Chinese hamster ovary cell

Multidrug resistance (MDR) severely compromises the efficacy of cancer chemotherapy in the clinic. It occurs when tumours, which may initially have been sensitive, become resistant to a variety of anti-cancer drugs. In experimental models this type of resistance is characterised by reduced sensitivity to a range of structurally unrelated chemotherapeutic agents with diverse subcellular targets. This spectrum of drugs includes many therapeutically important natural products and their semisynthetic congeners and includes the anthracyclins, the vinca alkaloids and the epipodophyllotoxins. *In vitro* studies show that MDR is accompanied by reduced intracellular drug accumulation mediated by increased drug efflux. Overexpression of a plasma membrane efflux pump, the p170 glycoprotein (P-gp), the product of the *mdr1* gene, has been demonstrated in many cells with the MDR phenotype and transfection of *mdr* cDNA can confer the MDR phenotype through P-gp overexpression (Endicott and Ling, 1989 and references therein). Inhibitors of P-gp can therefore resensitise MDR cells, and many studies have been conducted into the use of P-gp inhibitors as modulators of MDR (Wigler and Patterson, 1993).

Dipyridamole (DP), because of its known interaction with nucleoside transport (Plagemann *et al.*, 1988; Belt *et al.*, 1993), has been most extensively studied as an augmentor of antimetabolite cytotoxicity (Schmoll *et al.*, 1990; Goel and Howell, 1991 and references therein). Nevertheless, several studies have demonstrated the potentiation of other agents by DP. Ramu *et al.* reported in 1984 that DP increased the cytotoxicity of doxorubicin 15-fold in an MDR subline but only 2-fold in the parental cell line. Since then several studies have demonstrated the potentiation of a variety of MDR drugs by DP (reviewed by Goel and Howell, 1991). There is good evidence to show that this is mediated via an interaction of DP with P-gp. DP has been shown to increase the steady-state intracellular concentration and cytotoxicity of both vincristine and actinomycin D, and this was associated with the inhibition of drug efflux, to a far greater extent in MDR variants than the parental cells (Asoh *et al.*, 1989). Furthermore, DP inhibited the photoaffinity labelling of P-gp with [$^3$H]azidopine (Asoh *et al.*, 1989).

The mechanism of the interaction of DP with the cellular response to VP16 is less clear. VP16 (etoposide), an epipodophyllotoxin derivative inhibitor of topoisomerase II, is usually included in the MDR phenotype, although its transport characteristics and relationship to P-gp are not as well characterised as the anthracyclins or vinca alkaloids. DP has been shown to increase the intracellular concentration of VP16 in both *mdr*-transfected cells and parental cells but this was not associated with a synergistic increase in VP16 cytotoxicity (Shalinsky *et al.*, 1990). In other studies DP enhanced the cytotoxicity, as well as increasing the intracellular accumulation and decreasing the efflux, of VP16 in the drug-sensitive 2008 cell line (Howell *et al.*, 1989*a*, *b*). In order to investigate further the role of DP-P-gp interactions in the potentiation of VP16 cytotoxicity we have measured the effects of DP on the cellular pharmacology of VP16 in parental CHO-K1 cells and the MDR subline CHO-Adr$^r$. This cell line was derived by exposure to increasing concentrations of doxorubicin and has been well characterised (Hoban *et al.*, 1992). It has 5-fold reduced doxorubicin accumulation, associated with a 4-fold amplification of *mdr1* and elevated P-gp. It is cross-resistant to a variety of drugs of the MDR family, e.g. actinomycin D but not non-MDR drugs, e.g. BCNU. The classical MDR antagonist, verapamil, reversed both doxorubicin and vincristine resistance in CHO-Adr$^r$ cells. CHO-Adr$^r$ cells also have reduced topoisomerase II expression and changes in some glutathione-*S*-transferase levels. In contrast to the reported effects of verapamil on doxorubicin accumulation and cytotoxicity, strikingly similar effects of DP on VP16 accumulation, efflux and growth inhibition were observed in both the parental and the MDR cell line.

### Materials and methods

### *Drugs and chemicals*

VP16 and DP were obtained from Sigma, Poole, Dorset, UK, stock solutions were made by dissolving them in dry dimethyl sulphoxide (DMSO). [$^3$H]VP16 (1.1 Ci mmol$^{-1}$) in ethanol was obtained from Moravek Biochemicals, Brea, CA, USA.

### *Cell lines*

Parental CHO-K1 and the P-gp-overexpressing MDR subline CHO-Adr$^r$ (Hoban *et al.*, 1992) were a gift from Dr C Robson, Department of Surgery, University of Newcastle upon Tyne. The cell line is reported as stable and it was not necessary to re-expose CHO-Adr$^r$ cells to the selecting agent

Correspondence: NJ Curtin
Received 24 July 1995; revised 16 October 1995; accepted 20 November 1995

(doxorubicin) during routine culture. The cells were routinely grown in Hams F-10 (Gibco, Paisley, UK) containing 10% fetal calf serum (Globepharm, Esher, Surrey, UK) and were shown to be mycoplasma-free during regular monitoring. Both mutant and parental cells had a doubling time of 12–14 h.

### *Growth inhibition assays*

Cells were seeded at $1-1.5\times10^3$ cells per well in 100 $\mu$l of medium in replicate 96-well plates (leaving the outer wells with 100 $\mu$l of medium alone to minimise 'edge effect') and allowed to attach overnight. After 16–24 h the medium was replaced with that containing varying concentrations of VP16 with or without 10 $\mu$M DP in a final DMSO concentration of 0.1%, ten replicate wells were used for each drug concentration. A replicate plate was fixed as described below to obtain an estimate of the cell density at the start of the drug incubation. The plates were incubated for 72 h at 37°C before assaying for cell growth as described previously (Skehan *et al.*, 1990). Briefly, the 96-well plates were fixed in ice-cold 10% trichloroacetic acid (TCA) followed by ice-cold methanol, washed in tap water and air dried. The plates (together with the preincubation plate) were stained with 0.4% (w/v) sulphorhodamine B (Sigma) in 1% acetic acid (100 $\mu$l per well) for 30 min then rinsed three times in 1% acetic acid to remove unbound stain. Protein-bound stain was extracted using 100 $\mu$l of 10 mM Tris base (Sigma) per well. The optical density of the wells was read on a computer-interfaced MR700 microtitre plate reader (Dynatech, Billingshurst, West Sussex, UK) relative to an air blank using a 570 nm filter.

### *Measurement of [$^3$H]VP16 accumulation*

In order to mimic the conditions for growth inhibition as closely as possible the accumulation of VP16 was measured in complete medium rather than buffer. Cells were harvested without trypsin, using EDTA in phosphate-buffered saline (PBS), to avoid the proteolytic digestion of plasma membrane proteins. The cells were resuspended at $2\times10^7$ ml$^{-1}$ in complete medium with or without 10 $\mu$M DP. [$^3$H]VP16 was added to give a final concentration of 10 $\mu$g ml$^{-1}$ (17 $\mu$M: 1.1 mCi $\mu$M$^{-1}$) and mixed vigorously. The cells were agitated at room temperature and triplicate 50 $\mu$l (i.e. $10^6$ cells) samples were spun through 100 $\mu$l of silicone oil, specific gravity 1.028 (Dow-Corning 556:550 9:11; BDH, Merck, Lutterworth, Leicestershire, UK) overlaying 50 $\mu$l of 3 M potassium hydroxide in microfuge tubes (0.4 ml, BDH) at intervals. The tubes were capped and cut in the silicone layer; the lower portion, containing the cells lysed in potassium hydroxide, was placed in scintillation vials. The intracellular $^3$H was measured, following neutralisation of the cell lysate with 0.25 M acetic acid, by scintillation counting on an LKB-Wallac S1410 $\beta$-counter. Viable cell counts (trypan blue exclusion) were taken at intervals during the accumulation period.

### *Measurement of [$^3$H]VP16 retention*

Cells, harvested as described for accumulation experiments, were resuspended at $2\times10^7$ ml$^{-1}$ in complete medium containing 10 $\mu$g ml$^{-1}$ (17 $\mu$M) [$^3$H]VP16 (1.1 mCi $\mu$M$^{-1}$) and agitated for 20 min at room temperature. Triplicate 50 $\mu$l (i.e. $10^6$ cells) samples were spun through oil (as described above) to determine the initial intracellular VP16 concentration and the remaining cell suspension was pelleted and resuspended in an equal volume of fresh medium with or without 10 $\mu$M DP and mixed thoroughly. The cells were agitated at room temperature and triplicate 50 $\mu$l samples were spun through oil into 3 M potassium hydroxide to lyse the cells at intervals. The intracellular $^3$H was measured as described for the accumulation experiments. Viable cell counts (trypan blue exclusion) were taken at intervals during the efflux period.

### Results

### *Growth inhibition assays*

The effect of VP16, in the presence or absence of 10 $\mu$M DP, on the growth of parental and MDR CHO cells is shown in Figure 1. As expected, the CHO-Adr$^r$ cells were more resistant to VP16 than the parental CHO-K1 cells. On the basis of the concentration that inhibits cell growth to 50% of control growth ($IC_{50}$) data the CHO-Adr$^r$ cells were about 18 times more resistant to VP16 than CHO-K1 cells (Table I). Co-incubation with 10 $\mu$M DP caused a significant increase in the sensitivity of CHO-Adr$^r$ cells but only partially overcame the resistance to VP16. There was also a significant enhancement of VP16 growth inhibition by DP in the parental cells. A comparison of the dose enhancement factor ($DEF_{50}$), calculated from the ratio of the $IC_{50}$ for VP16 in the absence of DP to the $IC_{50}$ for VP16 in the presence of DP, indicated that there was a greater enhancement in the parental ($DEF_{50}$ = 4.63±2.75) than the MDR cells ($DEF_{50}$ = 2.47±0.83), however this difference was not significant.

### *Intracellular VP16 accumulation*

The intracellular accumulation of VP16 was measured in the two cell lines over 60 min (Figure 2). In the absence of DP, steady-state concentrations of VP16 were reached within the first 5 min of incubation in both cell lines. The MDR CHO-Adr$^r$ cells accumulated significantly less VP16 than the wild-type CHO-K1 cells. However, the intracellular VP16 concentration was only 28±5% less in CHO-Adr$^r$ cells than in CHO-K1 cells. In the presence of DP, VP16 continued to accumulate so that steady-state levels had still not been reached by 60 min. DP effectively increased VP16 content relative to controls in both cell lines (Table II). This effect

**Figure 1** The effect of VP16 and DP on the growth of CHO-K1 and CHO-Adr$^r$ cells. CHO-K1 (●, ○) and CHO-Adr$^r$ (▼, ▽) cells were grown in increasing concentrations of VP16 in the absence (●, ▼) or presence (○, ▽) of 10 $\mu$M DP for 72 h before staining with sulphurhodamine B and measuring the optical density (OD) at 570 nm. Each point represents the mean and each vertical bar the s.e.m. of three independent experiments.

**Table I** Growth inhibition: effect of 10 $\mu$M DP on CHO-K1 and CHO-Adr$^r$ cells

| | *$IC_{50}$ μM VP16* *CHO-K1* | *CHO-Adr$^r$* | *Fold resistant* |
|---|---|---|---|
| Control | 1.39±0.36 | 24.77±5.20 | 17.82±5.94 |
| +10 $\mu$M DP | 0.30±0.16 | 10.02±2.64 | |
| $DEF_{50}$ | 4.63±2.75 | 2.47±0.83 | |

$IC_{50}$ values (calculated from a computerised fit of the Hill equation) are the mean±s.d. of three independent experiments.

**Figure 2** The effect of DP on VP16 accumulation in CHO-K1 and CHO-Adr$^r$ cells. Accumulation of [$^3$H]VP16 in CHO-K1 (●, ○) and CHO-Adr$^r$ (▼, ▽) cells in the absence (●, ▼) or presence (○, ▽) of 10 $\mu$M DP. Uptake was initiated in cells in complete medium by the addition of [$^3$H]VP16 (10 $\mu$g ml$^{-1}$), samples were taken after 1, 5, 10, 20, 40 and 60 min as described in Materials and methods. Each point represents the mean and each vertical bar the s.e.m. of three independent experiments.

**Figure 3** The effect of DP on VP16 retention in CHO-K1 and CHO-Adr$^r$ cells. Retention of [$^3$H]VP16 in CHO-K1 (●, ○) and CHO-Adr$^r$ (▼, ▽) cells in the absence (●, ▼) or presence (○, ▽) of 10 $\mu$M DP. Cells were incubated in complete medium containing [$^3$H]VP16 (10 $\mu$g ml$^{-1}$), after 20 min samples were taken to determine the initial intracellular VP16 concentration and the remaining cells resuspended in fresh medium ± 10 $\mu$M DP with further samples being taken after 1, 5, 10 and 20 min as described in Materials and methods. Each point represents the mean and each vertical bar the s.e.m. of three independent experiments.

**Table II** Effect of 10 $\mu$M DP on intracellular VP16 accumulation

| *Time (min)* | *Intracellular VP16: fold increase by 10 μM DP* CHO-K1 | CHO-Adr$^r$ |
|---|---|---|
| 1 | 1.11 ± 0.18 | 1.44 ± 0.48 |
| 5 | 1.45 ± 0.14 | 2.02 ± 0.13 |
| 10 | 1.92 ± 0.14 | 2.37 ± 0.22 |
| 20 | 2.45 ± 0.9 | 3.00 ± 0.35 |
| 40 | 3.26 ± 0.43 | 3.79 ± 0.42 |
| 60 | 3.38 ± 0.37 | 4.04 ± 0.06 |

Figures are the mean ± s.d. (calculated as the intracellular [VP16] in the presence of 10 $\mu$M DP/the intracellular [VP16] in the absence of DP at each time point) from three independent experiments.

**Table III** Effect of 10 $\mu$M DP on retention of VP16

| *Time (min)* | *VP16 retention: fold increase with DP* CHO-K1 | CHO-Adr$^r$ |
|---|---|---|
| 1 | 1.63 ± 0.33 | 2.09 ± 0.63 |
| 5 | 2.75 ± 1.21 | 2.70 ± 1.06 |
| 10 | 2.80 ± 1.09 | 2.83 ± 0.69 |
| 20 | 2.59 ± 1.09 | 2.55 ± 0.81 |

Figures are the mean ± s.d. (calculated as the intracellular [VP16] in the presence of 10 $\mu$M DP/the intracellular [VP16] in the absence of DP at each time point) from three independent experiments.

was amplified with increasing incubation time so that by 40 min DP had increased the intracellular VP16 concentration 3.26 ± 0.43-fold in CHO-K1 cells and 3.79 ± 0.42-fold in CHO-Adr$^r$ cells. Overall, the effect of DP on the accumulation of VP16 by CHO-Adr$^r$ cells did not differ significantly from that on CHO-K1 cells.

*Intracellular retention of VP16*

The maintenance of lower steady-state intracellular [VP16] in the absence of DP than in the presence of DP suggested that either DP stimulates the uptake of VP16 or that there is an efflux mechanism operating to limit intracellular accumulation of VP16 that can be inhibited by DP. Therefore the effect of DP on [$^3$H]VP16 efflux in preloaded CHO-K1 and CHO-Adr$^r$ cells was investigated (Figure 3). The intracellular VP16 content was 8.3 ± 3 pmol $10^6$ CHO-K1 cells and 7.4 ± 3.9 pmol $10^6$ CHO-Adr$^r$ cells before efflux. In the absence of DP the initial efflux was very rapid in both cell lines (> 50% intracellular VP16 content lost in 1 min). Thereafter the rate of efflux was slower. DP retarded the efflux of VP16 in both cell lines, this effect was primarily on the initial rapid efflux phase. DP had a slightly greater effect on the CHO-Adr$^r$ cells at 1 min, but this was not significant and thereafter the magnitude of the effect mediated by DP was remarkably similar in both lines (Table III).

### Discussion

These studies show that the CHO-Adr$^r$ cell line is approximately 18-fold resistant to VP16. However, in drug accumulation studies the intracellular steady-state level of VP16 in CHO-Adr$^r$ cells was only 28% lower than in CHO-K1 cells, in contrast to the 5-fold reduced doxorubicin accumulation reported for these cells (Hoban *et al.*, 1992). This suggests that the resistance to VP16 in CHO-Adr$^r$ cells was mainly due to other mechanisms, probably the 4-fold reduction in topoisomerase II activity also reported for this cell line (Hoban *et al.*, 1992).

CHO-Adr$^r$ cells have been reported to have a 4-fold amplification of the *mdr1* gene and marked P-gp overexpression that results in a 5-fold increase in doxorubicin efflux in CHO-Adr$^r$ cells compared with the parental cells (Hoban *et al.*, 1992). However, VP16 retention is not significantly different in CHO-Adr$^r$ from that in CHO-K1 cells after 5 min in drug-free medium (Figure 3). These data suggest that P-gp plays only a modest role in the efflux of VP16. Additional evidence suggests that, although VP16 is recognised by P-gp, it is a poor substrate, for example in a study of 13 VP16-resistant CHO sublines none overexpressed P-gp (Soues *et al.*, 1995). Other studies have shown that verapamil, an MDR antagonist, does not increase the retention of VP16 in P-gp-overexpressing Chinese hamster lung cells (that accumulate 2- to 4-fold less VP16 than the parental cells) indicating that P-gp was not important in the efflux of VP16 (Politi *et al.*, 1990). Moreover, VP16 is a poor inhibitor of the photoaffinity labelling of P-gp by either azidopine (Sehested *et al.*, 1992) or verapamil (Politi *et al.*, 1990).

The effect of DP on the efflux of, the accumulation of and the sensitivity to VP16 was essentially the same in both CHO-K1 and CHO-Adr$^r$ cells. This is in marked contrast to the effect of verapamil, which has been shown to increase the accumulation of and sensitivity to doxorubicin to a far greater extent in CHO-Adr$^r$ cells than CHO-K1 cells (Chatterjee *et al.*, 1990; Hoban *et al.*, 1992). In these studies verapamil also augmented the accumulation and cytotoxicity of doxorubicin in the parental CHO-K1 cells to a small extent, presumably owing to the intrinsic moderate expression of P-gp in wild-type CHO cells (Gupta, 1988). Nevertheless, if DP was sensitising cells to VP16 via a P-gp mechanism, a greater effect on CHO-Adr$^r$ cells than CHO-K1 cells would be expected.

DP increased VP16 retention 2- to 3-fold, accumulation 3- to 4-fold and growth inhibition 2- to 4-fold, i.e. the magnitude of the effect of DP was similar on all three parameters. The implication is that the DP-mediated effect on growth inhibition is due to increased cellular exposure to VP16, which is related to the reduction in efflux. DP does appear to be acting via the inhibition of VP16 efflux but to the same extent in both cell lines. This implies that it is acting on an efflux mechanism that is not overexpressed in CHO-Adr$^r$ cells compared with the parental cells. Strikingly similar effects of DP on VP16 accumulation, retention and cytotoxicity have been reported by Howell *et al.* (1989*b*) for the drug-sensitive human ovarian carcinoma cell line, 2008. Furthermore these authors demonstrated that the synergy between DP and VP16 was not due to displacement of VP16 from serum proteins by DP. Similarly, Shalinsky *et al.* (1990) observed that DP increased the intracellular steady-state concentration of VP16 in cells that do not overexpress P-gp and concluded that DP could potentiate MDR drugs by both P-gp-dependent and P-gp-independent mechanisms.

The inhibition of VP16 efflux by a mechanism that is not overexpressed in CHO-Adr$^r$ cells (P-gp-independent) could be due to an effect of DP on the passive diffusion of VP16 across the plasma membrane. However, CHO-K1 and CHO-Adr$^r$ cells maintain lower intracellular concentrations of VP16 in the absence of DP than in its presence (Figure 2) and DP is known to interact with a variety of plasma membrane transporter proteins (e.g. nucleoside transporter, glucose transporter, P-gp). Thus, it is also possible that DP is inhibiting a VP16 efflux transporter protein that is expressed to the same extent in both CHO-K1 and CHO-Adr$^r$ cells. Recent studies (Soues *et al.*, 1995) using CHO sublines isolated by resistance to VP16 found that in two highly resistant sublines there was neither reduced topoisomerase II nor increased P-gp expression, suggesting the possible overexpression of another, unidentified, efflux transporter. Other studies demonstrate that novobiocin can potentiate VP16 cytotoxicity in non-P-gp-overexpressing multidrug-resistant cell lines, mediated through the intracellular accumulation of VP16, but in P-gp-overexpressing cell lines novobiocin increased neither the intracellular accumulation nor the cytotoxicity of VP16 (Rappa *et al.*, 1993).

It is now becoming clear that P-gp is not the only plasma membrane drug efflux protein. At least one other protein, the multidrug resistance protein, MRP (Cole *et al.*, 1992), also confers resistance to natural product anti-cancer drugs. There is evidence to suggest that, whereas VP16 is a poor substrate for P-gp (Politi *et al.*, 1990; Sehested *et al.*, 1992; Soues *et al.*, 1995), it is a good substrate for MRP, namely in MCF7 cells made resistant to VP16 MRP mRNA was increased at least 10-fold (Schneider *et al.*, 1994) and in two HeLa sublines transfected with MRP the resistance to VP16 (11.6- and 8.9-fold) was greater than that for most of the other drugs evaluated (Cole *et al.*, 1994). It is tempting to speculate that DP augmentation of VP16 in CHO-K1 and CHO-Adr$^r$ cells may be mediated through inhibition of MRP. CHO-K1 cells have a modest overexpression of P-gp (Gupta, 1988) before selection for drug resistance: it is possible that they might also express MRP and that this level of expression was not increased during the selection for resistance to doxorubicin. However, in a VP16-resistant human ovarian cell line neither P-gp nor MRP overexpression was detected despite reduced drug accumulation in these cells (Kubota *et al.*, 1994), which might indicate the existence of yet another drug efflux protein that could be a target for DP. Future studies using an MRP-overexpressing line will be necessary to address this question.

**Abbreviations**

MDR, multidrug resistance; DP, dipyridamole; P-gp, P-glycoprotein; DMSO, dimethyl sulphoxide; TCA, trichloroacetic acid; $IC_{50}$, concentration that inhibits cell growth to 50% of control growth; $DEF_{50}$, dose enhancement factor at the $IC_{50}$.

**Acknowledgements**

We gratefully acknowledge the support of The North of England Children's Cancer Research Fund (RN Turner) and The North of England Cancer Research Campaign (NJ Curtin).

### References

ASOH K-I, YOSHIO S, SATO S-I, NOGAE I, KOHNO K AND KUWANO M. (1990). Potentiation of some anticancer agents by dipyridamole against drug-sensitive and drug-resistant cancer cell lines. *Jpn. J. Cancer Res.*, **80**, 475–481.

BELT JA, MARINA NM, PHELPS DA AND CRAWFORD CR. (1993). Nucleoside transport in normal and neoplastic cells. *Adv. Enzyme Regul.*, **33**, 235–252.

CHATTERJEE M, ROBSON CN AND HARRIS AL. (1990). Reversal of multidrug resistance by verapamil and modulation by $\alpha_1$-acid glycoprotein in wild-type and multidrug-resistant Chinese hamster ovary cell lines. *Cancer Res.*, **50**, 2818–2822.

COLE SPC, BHARDWAJ G, GERLACH JH, MACKIE JE, GRANT CE, ALMQUIST KC, STEWART AJ, KURZ EU, DUNCAN AMV AND DEELEY RG. (1992). Overexpression of a transporter gene in a multidrug-resistant human lung cancer cell line. *Science*, **258**, 1650–1654.

COLE SPC, SPARKS KE, FRASER K, LOE DW, GRANT CE, WILSON GM AND DEELEY RG. (1994). Pharmacological characterisation of multidrug resistant MRP-transfected human tumour cells. *Cancer Res.*, **54**, 5902–5910.

ENDICOTT JA AND LING V. (1989). The biochemistry of P-glycoprotein-mediated multidrug resistance. *Annu. Rev. Biochem.*, **58**, 137–171.

GOEL R AND HOWELL SB. (1992). Modulation of the activity of cancer chemotherapeutic agents by dipyridamole. In *New Drugs, Concepts and Results in Cancer Chemotherapy*, Muggia FM. (ed.) pp. 19–44. Kluwer Academic Press: Boston, MA.

GUPTA RS. (1988). Intrinsic multidrug resistance phenotype of Chinese hamster (rodent) cells in comparison to human cells. *Biochem. Biophys. Res. Comm.*, **153**, 598–605.

HOBAN PR, ROBSON CN, DAVIES SM, HALL AG, CATTAN AR, HICKSON ID AND HARRIS AL. (1992). Reduced topoisomerase II and elevated $\alpha$class glutathione *S*-transferase expression in a multidrug resistant CHO cell line highly cross-resistant to mitomycin C. *Biochem. Pharmacol.*, **43**, 685–693.

HOWELL SB, HOM D, SANGA R, VICK JS AND ABRAMSON IS. (1989*a*). Comparison of the synergistic potentiation of etoposide, doxorubicin and vinblastine cytotoxicity by dipyridamole. *Cancer Res.*, **49**, 3178–3183.

HOWELL SB, HOM D, SANGA R, VICK JS AND CHAN TKC. (1989*b*). Dipyridamole enhancement of etoposide sensitivity. *Cancer Res.*, **49,** 4147–4153.

KUBOTA N, NISHIO K, TAKEDA Y, OHMORI T, FUNAYAMA Y, OGASAWARA H, OHIRA T, KUNIKANE H, TERASHIMA Y AND SAIJO N. (1994). Characterisation of an etoposide-resistant human ovarian cancer cell line. *Cancer Chemother. Pharmacol.*, **34,** 183–190.

PLAGEMANN PGW, WOHLHUETER RM AND WOFFENDIN C. (1988). Nucleoside and nucleobase transport in animal cells. *Biochim. Biophys. Acta.*, **947,** 405–443.

POLITI PM, ARNOLD ST, FELTSED RL AND SINHA BK. (1990). P-glycoprotein-independent mechanism of resistance to VP-16 in multidrug-resistant tumour lines: Pharmacokinetic and photo-affinity labelling studies. *Mol. Pharmacol.*, **37,** 790–796.

RAMU A, SPANIER R, RAHAMIMOFF H AND FUKS Z. (1984). Restoration of responsiveness in doxorubicin-resistant P388 murine leukaemia cells. *Br. J. Cancer*, **50,** 501–507.

RAPPA G, LORICO A AND SARTORELLI AC. (1993). Reversal of etoposide resistance in non-p-glycoprotein expressing multidrug resistant tumour cells by novobiocin. *Cancer Res.* **53,** 5487–5493.

SCHMOLL H-J, HARSTRICK A, KOHNE-WOMPNER C-H, SCHOBER C, WILKE H AND POLIWODA H. (1990). Modulation of cytotoxic drug activity by dipyridamole. *Cancer Treat. Rev.*, **17,** (suppl. A) 57–65.

SCHNEIDER E, HORTON JK, YANG C-H, NAKAGAWA N AND COWAN KH. (1994). Multidrug resistance-associated protein gene overexpression and reduced drug sensitivity of topoisomerase II in a human breast carcinoma MCF7 cell line selected for etoposide resistance. *Cancer Res.*, **54,** 152–158.

SEHESTED M, FRICHE E, JENSEN PB AND DEMANT EJF. (1992). Relationship of VP-16 to the classical multidrug resistance phenotype. *Cancer Res.*, **52,** 2874–2879.

SHALINSKY DR, ANDREEFF M AND HOWELL SB. (1990). Modulation of drug sensitivity by dipyridamole in multidrug resistant tumour cells *in vitro*. *Cancer Res.*, **50,** 7537–7543.

SKEHAN P, STORENG R, SCUDIERO D, MONKS A, MCMAHON J, VISTICA D, WARREN JT, BOKESCH H, KENNEY S AND BOYD MR. (1990). New colorimetric cytotoxicity assay for anticancer-drug screening. *J. Natl Cancer Inst.*, **82,** 1107–1112.

SOUES S, LAVAL F AND CHARCOSSET J-Y. (1995). Mechanism of resistance to combinations of vincristine, etoposide and doxorubicin in Chinese hamster ovary cells. *Br. J. Cancer*, **71,** 489–497.

WIGLER PW AND PATTERSON FK. (1993). Inhibition of the multidrug resistance efflux pump. *Biochim. Biophys. Acta.*, **1154,** 173–181.